# Recurrent convolutional neural kernel model for stock price movement prediction

**DOI:** 10.1371/journal.pone.0234206

**Published:** 2020-06-12

**Authors:** Suhui Liu, Xiaodong Zhang, Ying Wang, Guoming Feng

**Affiliations:** Donlinks School of Economics and Management, University of Science and Technology Beijing, Beijing, China; Shandong University of Science and Technology, CHINA

## Abstract

Stock price movement prediction plays important roles in decision making for investors. It was usually regarded as a binary classification task. In this paper, a recurrent convolutional neural kernel (RCNK) model was proposed, which learned complementary features from different sources of data, namely, historical price data and text data in the message board, to predict the stock price movement. It integrated the advantage of technical analysis and sentiment analysis. Different from previous studies, the text data was treated as sequential data and utilized the RCNK model to train sentiment embeddings with the temporal features. Besides, in the classification section of the model, the explicit kernel mapping layer was used to replace several full-connected layers. This operation reduced the parameters of the model and the risk of overfitting. In order to test the impact of treating the sentiment data as sequential data, the effectiveness of explicit kernel mapping layer and the usefulness integrating the technical analysis and sentiment analysis, the proposed model was compared with the other two deep learning models (recurrent convolutional neural network model and convolutional neural kernel model) and the models with only one source of data as input. The result showed that the proposed model outperformed the other models.

## Introduction

Stock price movement prediction has long been a hot issue in both academic and industrial fields. Accurate prediction can not only help shareholders to make appropriate decisions and obtain excess return but also can be an important indicator for stock portfolio selection [1, 2]. The efficient market hypothesis (EMH) [3] presumes that all the participants in the market are informationally efficient and all deals are traded in fair values. In other words, the stock market responds quickly and accurately to new market information and the stock price fully reflect all information, thus the stock market is unpredictable. Nevertheless, the presumption in the EMH is not practical in real world. The adaptive market hypothesis (AMH) [4] is proposed by behavioural economists to reconcile the EMH. The AMH considers the interaction of the market and participants and holds that the excess return comes from information asymmetry, which means the stock market is predictable. Besides, with the advent of information overload, a new theory, heterogeneous agent models (HAM) [5], comes out. It holds that the ability to quickly utilize and mine the information varied among participants. Thus, stock cycles still appear in efficient market [6]. Usually, predicting the direction of stock price movement is regarded as a binary classification task which consists of two stages: data representation and classification. A lot of effort has been focused on the optimization of either of them. The classical and state-of-art works for these two aspects will be introduced in the following parts.

Theoretically, the more comprehensive the market information we have and the more reasonable our utilization of the information, the more likely we are able to get better prediction results. Exploring financial time series data and modelling the correlation between historical data and the stock trend is a primary way to solve the problem [7–9]. Historical price data and trading volumes were used to form some technical indicators (TIs) as feature inputs to predict stock trend. The TIs describe additional information such as the trend of stock price movement, whether the stock is oversold or overbought and so on. This approach is also known as technical analysis (TA). Due to the convenience for data representation and the learning power of deep learning, it is still a popular method for stock price prediction. However, the focus of researches has gradually shifted to how to build new or hybrid deep learning models to learn more features from TIs and financial time series data other than designing new technical indicators. Wen et al. [10] proposed a multi-filter neural network (MFNN) to extract features from historical price data, which outperformed traditional machine learning models. Hoseinzade and Haratizadeh [11] constructed a convolutional neural network (CNN) based frame work for feature extraction on TIs and financial time series data from a variety of sources. Kim and Kim [12] used a feature fusion LSTM-CNN model to learn different features from different representation of the same financial time series data. The long-short term memory (LSTM) part was used to learn temporal features from numerical financial time series data and the CNN part was used to learn image features from the same data that were represented by stock chart images. Qiu, Wang and Zhou [13] added attention mechanism to LSTM neural network to enhance predictive power of the model.

However, according to the literature [14], the correlation between historical data and stock return receded. It is inadequate to predict stock price movement by technical analysis alone. Especially with the development of the Internet and social media, a large number of valuable text information such as financial news, reports and user comments emerge, which has a significant impact on the stock market. This puts forward a new demand for forecasting stock price movement: how to extract key information from massive text data. Fortunately, the rapid development of natural language processing (NLP) makes it possible to automatically read numerous texts and measure their impact on stock price movement. A considerable number of researchers have concentrated on text-based stock price movement prediction via machine learning techniques [15–19]. They sought to use sentiment analysis (SA) to study whether the texts published in a period of time were good or bad for the stock market, so as to predict the stock price movement. One of the main parts for sentiment analysis is to extract key sentiment information from raw text data and convert them into standard formats that can be directly fed to models. After that, the text will be represented by a vector each of whose dimensions correspond to a specific term. Li et al. [16] used the Harvard psychological dictionary and Loughran-McDonald financial sentiment dictionary to construct a six-dimensional sentiment space. They filtered each piece of financial news with these two dictionaries and accumulated the sentiment phrases together to form a sentiment embedding as an input feature for the prediction model. Si et al. [20] utilized a continuous Dirichlet Process Mixture model to learn the daily topic set from Twitter and derived its sentiment according to the opinion words distribution. They regressed the Twitter sentiment and the stock index to predict the market. Nguyen, Shirai and Velcin [21] proposed a Topic Sentiment Latent Dirichlet Allocation (TSLDA) model to calculate the sentiment values of topic-sentiment pairs as a novel feature for stock prediction. They extracted consecutive nouns in sentences as a topic and utilized SentiWordNet [22] to extract opinion words and accumulated sentiment value of opinion words for each topic. However, the text representation mentioned above cannot capture the semantic similarity when expressing one meaning by different phrases, which may cause the dimension explosion. In addition, the quality of text representation relies deeply on man-made tricks to select features. With the development of neural network-based language model [23, 24], a new text representation, word embedding, has emerged to overcome these disadvantages. It transforms a word into a dense and fixed-dimensional vector which is a distributed and compact representation of the word [25]. Each dimension of the word embedding could be regarded as an abstract semantic feature and the similarity of two words could be assessed by the cosine of angles between these two embeddings. Usually, word embedding representation is accompanied by deep learning classification model. Ding et al. [26] used convolutional neural networks (CNN) to train event embeddings at the base of word embedding representation for stock price movement prediction. Peng and Jiang [27] applied the word embedding methods and deep neural networks to leverage financial news to predict stock price movements. The results had shown that it can significantly improve the prediction accuracy on a standard financial database over the baseline system using only the historical price data. Xu et al. [28] proposed a recurrent convolutional neural network (RCNN) to extract key features and context-dependent relations in financial news to predict the stock movement. It can achieve improvement in individual stock prediction. Vargas et al. [29] also proposed a similar recurrent convolutional neural network (RCNN) for predicting the direction of stock price movement, but it extracted features from both financial news and financial time series data. Yang et al. [30] proposed a dual-layer attention-based neural network and applied sentence embedding and day embedding for stock prediction. The model paid more attention to the more influential news and showed much better explainable result than traditional neural networks.

As for the classification section of the model, according to the literature [31], supporting vector machine (SVM) was the most popular classification model among previous researches on stock forecasting. As the most commonly used kernel method, it has a firm mathematical foundation. By the non-linear kernel function, input vectors can be implicitly projected into a high dimensional space so that a hyperplane could be constructed to separate the inputs linearly. However, the accuracy of prediction is largely dependent on the quality of input features as well as the selection of kernel function and pre-defined parameters. In contrast, the neural network model can automatically learn the advanced features from input data for prediction. Especially the recurrent neural network (RNN) model can extract context-feature or temporal features from sequential data [24]. Convolutional neural network (CNN) can also learn effective features for text classification [32, 33]. The results of deep learning models were not less than those of traditional machine learning methods. But the neural network also has the disadvantage that cannot be ignored. The gradient will disappear or explode with the increase of neural network layers, which makes the model unable to update. In addition, with the increase of parameter numbers, the overfitting problem is easy to occur, which makes the performance of the model on the test set very poor. Therefore, it is better to construct a hybrid model, which combines the advantages of both the kernel method and neural network models and overcomes their shortcomings. Some studies have attempted to combine these two methodologies. Le and Xie [34] proposed a deep embedding kernel (DEK) and test the performance on serval classification tasks. The DEK utilized deep learning to train a kernel, which in turn implicitly mapped data to a high dimensional feature space. In other words, the DEK applied the core idea of the kernel method on deep learning models and regarded the output of DEK model as the output of kernel function. It showed good performance on predicting stock daily price direction with financial series data. Mehrkanoon [35,36] and Suykens [35] introduced another hybrid deep neural kernel framework which replaced the activation function of neural networks by random Fourier feature maps. The results showed an improvement of classification accuracy over standard neural networks on several real-life digits or images datasets. However, none of these hybrid models has been applied on text classification or considered the features from text data. Compared with numerical financial time series data and images, text data is more difficult to be processed and understood by computers. In this paper, we tried to build a new hybrid model that can learn features from financial time series data as well as text data.

Our motivation is to build a model that combines technical analysis and sentiment analysis. In our proposed model, the temporal feature of sentiment in text data will be considered and the classification section of the model will be optimized to improve the accuracy for stock price movement prediction. We used the historical stock price data and posts in the message board as two sources of datasets. Historical price data give an objective description of a stock’s performance and the posts in the message board represent the subjective public mood towards the stock, thus exploring these two sources of data could generate complementary features and bring improvement for prediction. Furthermore, we assumed that the influence of the public mood on the stock price would last for a period and gradually recede over time. Thus, we aimed to build a model that can learn the temporal features of sentiment data. In this paper, we proposed a model named Recurrent Convolutional Neural Kernel (RCNK) model to train sentiment embedding with temporal and contextual features extracted from user comments. It is a hybrid model of neural network and kernel method. In the classification section of the model, we utilized the explicit kernel mapping layer to replace the full-connected layers in traditional neural networks. It maps the feature vectors into a new space where the vectors could be linearly separable. This technique decreased the number of neural network layers and improved the prediction accuracy of the model.

## Problem formalization

Stock price movement prediction is regarded as a classification task. As described in Eq 1, it is assigned a label for each transaction date of the stock *i*. If the closing price *pc*_*t*_ on the transaction date *t* is higher than that on the transaction date *t*−1, the sample would be labelled 1, otherwise 0.

$$l_{i}(t)=\left\{ \begin{array}{c} 0,\;{pc}_{t}<{pc}_{t-1}\\ 1,\;{pc}_{t}\geq {pc}_{t-1} \end{array}\right.$$ (1)

It is aimed to predict the right label of a stock by analysing two types of input data. The first part is the financial time series data. It denotes a sequence of vectors:

$$\mathrm{Input}\_1_{i}(t)=\left[T_{t-m}^{i},T_{t-m+1}^{i},\ldots \ldots T_{t-1}^{i}\right]$$ (2)

where vector $T_{t}^{i}$ includes the stock price and technical indicators on transaction date *t* of the stock *i*. The second part is a sequence of user-generated posts within m days before the transaction date *t*. It is denoted as:

$$\mathrm{Input}\_2_{i}(t)=\left[S_{t-m}^{i},S_{t-m+1}^{i},\ldots \ldots S_{t-1}^{i}\right]$$ (3)

where $S_{t}^{i}$ denotes a collection of selected top posts at date *t* of the stock *i*. How to transform the text data into numerical vectors will be described in detail in data preparation section. The output *y*_*i*_(*t*) of our model is the prediction result. It represents the probability that the stock price will rise at date *t*, which is computed as:

$$y_{i}\left(t\right)=F(Input\_1_{i}\left(t\right),\;Input\_2_{i}\left(t\right))$$ (4)

where *F*(∙)will be described in detail in model design section. The learning objective of our model is to minimize the cross-entropy loss. It is computed as:

$$\min\;loss=min\underset{N}{\sum }\underset{T}{\sum }[l_{i}\left(t\right)*logy_{i}\left(t\right)+\left(1-l_{i}\left(t\right)\right)*\log\left(1-y_{i}\left(t\right)\right)]$$ (5)

where *N* denotes the numbers of stocks, *T* denotes the numbers of transaction date. In this study, the random gradient descent algorithm and back propagation were utilized to find out the most optimal parameters.

## Data preparation

**Fig 1:**
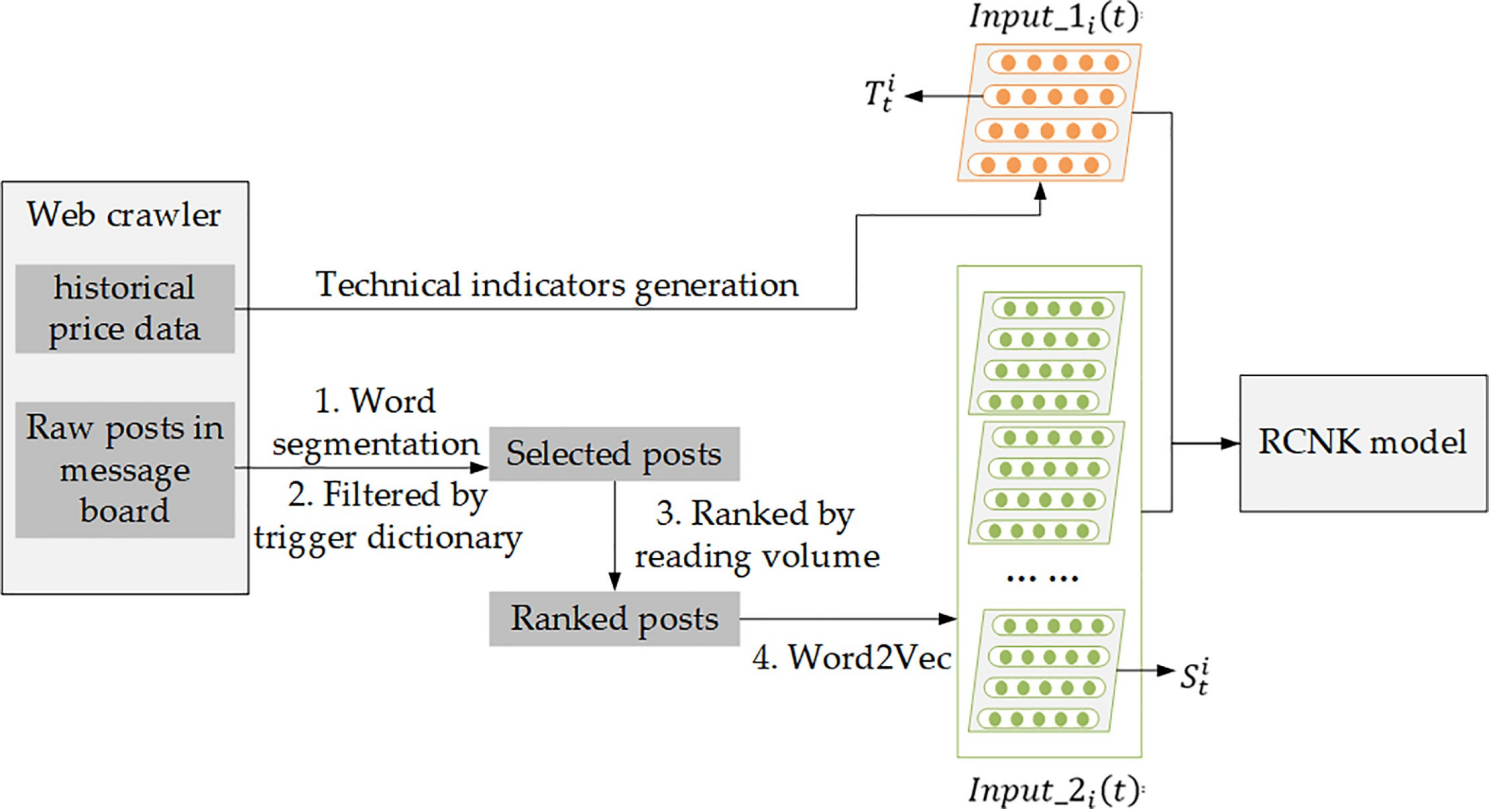
The process of data preparation.

The datasets were all crawled from http://guba.eastmoney.com, which is a well-known website about stocks in China. It not only displays real-time trading information, but also provides a platform for users to comment on the stock. The collection method complied with the terms and conditions for the website http://guba.eastmoney.com. The data did not contain any identifying information, and they were accessed from publicly available posts. The process of data preparation is illustrated in Fig 1.

The message board is a platform for stock investors to share their experience, discuss stock market quotations and vent their emotions. Thus, the themes of the posts are varied and it was necessary to select posts that are meaningful for prediction. In theory, the more meaningful the posts are, the higher the reading volumes are. Besides, posts with objective analysis of events and stock performance have a greater impact on the accuracy of stock price movement prediction than that only with subjective wishes or extreme emotions. Thus, after word segmentation, a trigger dictionary containing technical terms of stock markets to filter posts with effective features and a stop-word vocabulary to block posts that vented extreme negative emotions were used. After filtering and statistics, it was found that the mode of the number of comments per stock per day was 20. Thus, those posts were ranked according to the reading volume and the top 20 posts were selected for each stock on each transaction date (padding with zero vectors if there were less than 20 posts).

Word embeddings have been pre-trained with Word2Vec tools. After word segmentation and elimination of stop words, most of the posts have no more than 10 words. Considering the maximum retention of semantic features and computational complexity, it was decided to represent each post as the average of the word embeddings of the first 10 words. Finally, the *Input*_2_*i*_(*t*) was transformed into a sequence of word embedding matrixes. Each matrix $S_{t}^{i}$ represents the selected posts for stock *i* on transaction date *t*. Each row of $S_{t}^{i}$ is an average of word embeddings of a post. In other words, each row of $S_{t}^{i}$ represents a post for stock *i* on transaction date *t*.

As for *Input*_1_*i*_(*t*), the time series data used mainly include stock historical data and technical indicators as input features. The most commonly used historical data are open price, close price, highest price, lowest price, trading volume, etc. The technical indicators describe additional information derived from stock price in different ways. They include moving average (MA), rate of change (ROC), relative intensity index (RSI), Williams indicator and so on. The input features have been described in Table 1.

**Table 1:** Input features in *Input*_1_*i*_(*t*).

Feature	Formula	Description
*po*_*t*_	-	Stock open price at date *t*
*pc*_*t*_	-	Stock close price at date *t*
*ph*_*t*_	-	Highest stock price at date *t*
*pl*_*t*_	-	Lowest stock price at date *t*
*v*_*t*_	-	The number of shares traded at date *t*
*SMA*_*n*_(*pc*)	$\frac{1}{n}\sum_{i=0}^{n-1}pc(t-i)$	A price trend indicator calculated as the average of stock close price over *n* days
*EMA*_*n*_(*pc*)	α*pc_*t*_+(1−*α*) *EMA*_*n*−1_(*pc*), $\alpha =\frac{2}{1+\Delta },\;\Delta =time\;span$	A price trend indicator calculated as the exponential average of stock close price over *n* days
*TR*	max(*ph*_*t*_−*pl*_*t*_,*ph*_*t*_−*pc*_*t*−1_,*pc*_*t*−1_−*pl*_*t*_)	A price volatility indicator at date *t*
*ATR*_*n*_	*EMA*_*n*_(*TR*)	A price volatility trend indicator over *n* days
*RSI*_*n*_	100−100(*EMA*_*n*_(*M*^+^)/*EMA*_*n*_(*M*^−^)), *M*^+^ = max(*pc*_*t*_−*pc*_*t*−1_,0), *M*^−^ = min(*pc*_*t*_−*pc*_*t*−1_,0)	Relative strength index of price trend over *n* days that compares the magnitude of recent gains to recent losses
*ROC*_*n*_	(*pc*_*t*_−*pc*_*t*−*n*_)/*pc*_*t*−*n*_	Price rate-of-change indicator
*William*_*R*_*n*_	$100*\frac{{max(ph}_{n})-{pc}_{t}}{{max(ph}_{n})-{min(pl}_{n})}$	A momentum indicator that shows the relationship between the current close price and highest, lowest price over past *n* days
*Stochastic*_*K*_*n*_	$100*\frac{{pc}_{t}-{min(pl}_{n})}{{max(ph}_{n})-{min(pl}_{n})}$	A momentum indicator that gives a signal meaning that a stock is oversold or overbought
*Stochastic*_*D*_*n*_	*EMA*_3_(*Stochastic*_*K*_*n*_)	Exponential moving average of *Stochastic*_*K*_*n*_
*CCI*_*n*_	$\frac{M_{t}-{SMA}_{n}(M_{t})}{0.015\sum_{i=1}^{n}\left|M_{t-i+1}-{SMA}_{n}(M_{t})\right|/n}$, *M*_*t*_ = *pc*_*t*_+*pl*_*t*_+*ph*_*t*_)	An oscillator indicator used to indicate whether a stock is oversold or over overbought

## Model design

In this section, we will introduce our proposed model named Recurrent Convolutional Neural Kernel model (RCNK) to predict stock price movement. It is a hybrid model that combines the advantages of the kernel method and deep learning.

**Fig 2:**
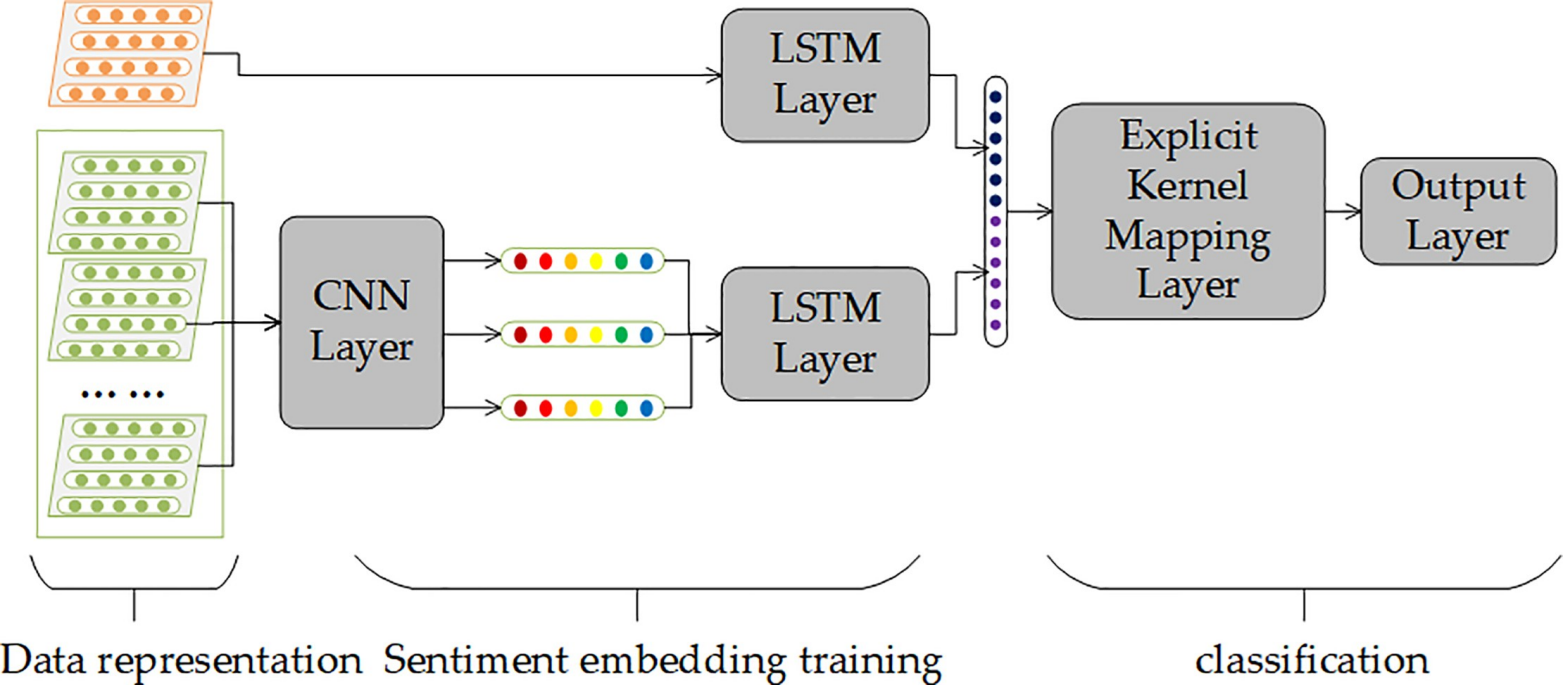
The architecture of Recurrent Convolutional Neural Kernel (RCNK) model.

The architecture of the model is shown in Fig 2. From the perspective of the task, it can be divided into three parts: data representation, sentiment embedding training and classification; from the perspective of model structure, it can be divided into five layers: input layer, convolutional neural network (CNN) layer, long-term and short-term memory (LSTM) neural network layer, an explicit kernel mapping layer and the output layer. These layers will be described as following in detail.

### CNN layer

The convolutional neural network has long been used in computer vision and achieved remarkable results. In recent years, it had also proven to be useful in natural language processing especially in text classification [24,25]. Hence, in this study, CNN layer was used to extract daily sentiment features from posts matrix $S_{t}^{i}$.

**Fig 3:**
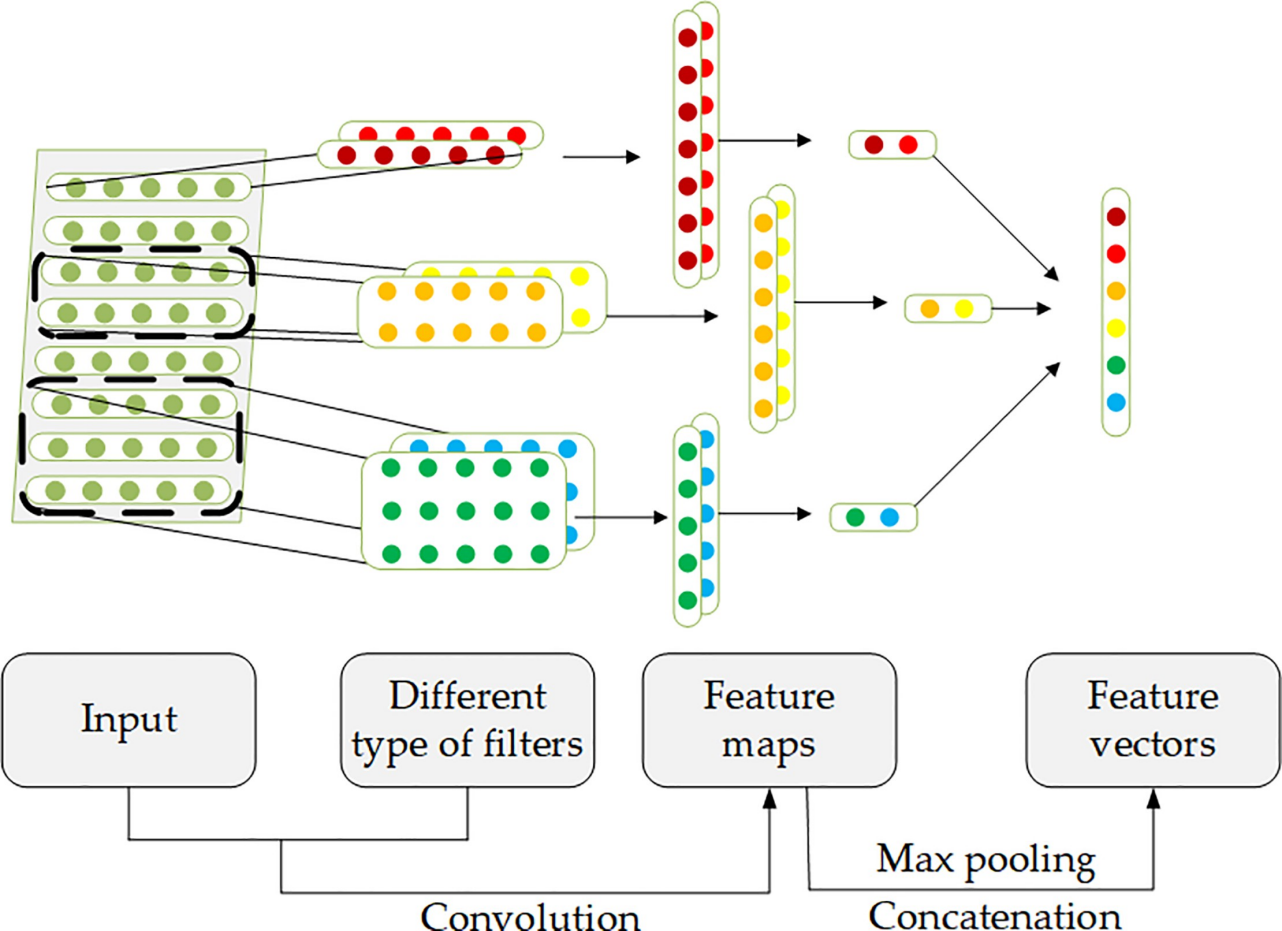
The processing of CNN layer.

As is shown in Fig 3, the filter strides along the matrix to produce a feature map whose elements are denoted by *a*_*i*,*j*_. Specifically, *x*_*i*,*j*_ denotes the element of *i*-th row, *j* -th column of $S_{t}^{i}$ and the *w*_*m*,*n*_ denotes the weight of *m*-th row, *n*-th column of filter. The feature *a*_*i*,*j*_ is computed according to Eq 6.

$$a_{i,j}=f(\sum_{m=0}^{M-1}\sum_{n=0}^{N-1}w_{m,n}x_{i+m,j+n}+w_{b})$$ (6)

where *M*, *N* ∈ R represent the length and width of the filter respectively, *w*_*b*_ ∈ R is a bias term and *f* is a non-linear activation function such as the hyperbolic tangent, rectified linear unit or sigmoid. Because each row of $S_{t}^{i}$ describes the abstract semantic features for one piece of post, it is reasonable to set *N* to be equal to the width of input matrix. As for *M* (also called window size), it usually has multiple values so extract combinatorial features could be extracted. It is noteworthy that the sentiment embedding is not trained for one post but for the totally selected posts in one day. In other words, the feature map *a*_*i*,*j*_ represents the comprehensive emotion of investors in the day *t*. Therefore, multiple values of *M* can help to extract more combinatorial features. Next, a max-pooling operation was applied to the feature map. It takes the maximum value of the feature map to form a new vector. This operation is to capture the most important feature, which is the highest value, for each input matrix. It can also reduce the risk of overfitting by cutting down some parameters [11]. Obviously, it can naturally deal with varying length of feature maps.

To sum up, one filter extracts one feature for one input matrix after the convolution and max-pooling operation. The CNN layer uses multiple filters with varying window sizes to obtain multiple features. These features are concatenated at the end of this layer. It is worth mentioning that the weights of filters are shared among the input matrix $S_{t}^{i}$ in *Input*_2_*i*_(*t*).

### LSTM layer

As it was mentioned above, *Input*_2_*i*_(*t*) is a sequential of text data, which describes investors’ sentiment information about the stock within several days before transaction date *t*. These sentiment information does not exist independently but has an impact on subsequent sentiment and weakens over time. Therefore, the purpose of this layer is to learn the continuous impact of previous sentiment on stock price on transaction date *t*. This impact can be called temporal features.

**Fig 4:**
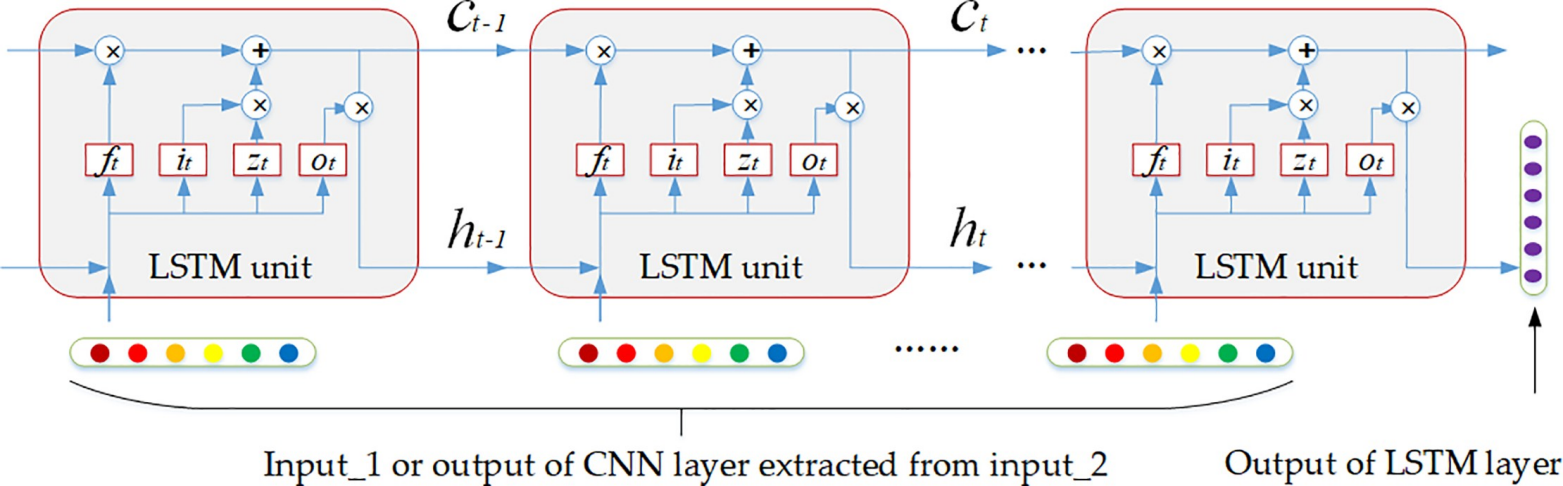
The processing of LSTM layer.

As is shown in Fig 4, LSTM unit uses a memory cell *C*_*t*_ and three gates to control the update of hidden state. The forget gate *f*_*t*_ decides which values of the previous memory cell *C*_*t*−1_ should be reserved for the current memory cell *C*_*t*_. The input gate *I*_*t*_ decides which values of input should be passed to the current memory cell *C*_*t*_. Finally, the output gate *O*_*t*_ controls which values of cell state *C*_*t*_ will have a further impact on the next time step. Thus, long-term and short-term memory neural network is effective in capturing temporal features on sequential data. The LSTM transition equations are following:

$$i_{t}=sigmoid(W_{i}x_{t}+U_{i}h_{t-1}+b_{i})$$ (7)

$$f_{t}=sigmoid(W_{f}x_{t}+U_{f}h_{t-1}+b_{f})$$ (8)

$$o_{t}=sigmoid(W_{o}x_{t}+U_{o}h_{t-1}+b_{o})$$ (9)

$$z_{t}=sigmoid(W_{z}x_{t}+U_{z}h_{t-1}+b_{z})$$ (10)

$$c_{t}=i_{t}\Theta z_{t}+f_{t}∙c_{t-1}$$ (11)

$$h_{t}=o_{t}\Theta \tanh\left(c_{t}\right)$$ (12)

where *x*_*t*_ is the input at current time step, sigmoid and tanh are non-linear activation function, *Θ* denotes element wise multiplication, *W*_*i*_, *W*_*f*_, *W*_*o*_, *W*_*z*_, *U*_*i*_, *U*_*f*_, *U*_*o*_, *U*_*z*_ are the weight matrixes and *b*_*i*_, *b*_*f*_, *b*_*o*_, *b*_*z*_ are the bias terms. The outputs of LSTM layer are the sentiment embedding with temporal features from *Input*_2_*i*_(*t*) and feature vector extracted from *Input*_1_*i*_(*t*). They will be concatenated together and passed on for classification layer.

### Explicit kernel mapping layer

In general, several fully-connected layers will be stacked over the feature extraction layer to get the probability distribution of classification. This operation brings a large number of parameters hence increases the computation complexity and the risk of overfitting problem. It is necessary to find a way to reduce the number of parameters and remain the accuracy of the prediction model at the same time. Inspired by [37], an explicit kernel mapping layer was constructed to replace the fully-connected layers. This is an approximate computation of kernel method. Due to the random projection process of constructing explicit mapping function *Z*(*x*), this layer does not bring any trainable parameters. Besides, it can bridge the deep learning model and the kernel method, which enables us to stack a mapping layer over the neural networks to achieve the best of two methodologies.

The common kernel method Support Vector Machine (SVM) applies a kernel function implicitly mapping the input features into a high-dimensional Hilbert space where the mapped data could be separated linearly. The simplicity of this method is that it is unnecessary to compute the coordinates of the mapped data but rather simply to compute the inner products of all pairs of data in the original space. Unfortunately, with the increase of training examples, the training speed of this method will decrease significantly. Rahimi and Recht [37] aimed to construct the explicit mapping function *Z*(*x*) that mapped the points in the original space to a relatively low-dimensional randomized feature space which is a lower-dimensional space compared with Hilbert space. This operation makes the inner product between a pair of transformed points approximating to their kernel evaluation. They proved their method could compete favourably in speed and accuracy with the kernel-based algorithms. The detailed process of constructing *Z*(*x*) is described as following.

This approach is inspired by Bochner’s theorem that if the kernel *k*(*δ*) is a positive definite shift-invariant kernel and is properly scaled, its Fourier transform *p*(*ω*) is a proper probability distribution. If we define *ζ*_*ω*_(*x*) = *e*^*jω*′*x*^, we will get

$$k(x,y)=k(x-y)=\int_{R^{d}}p(\omega )e^{j\omega^{\prime }(x-y)}d\omega =E_{\omega }[\zeta_{\omega }(x)\zeta_{\omega }(y)]$$ (13)

After transforming two points *x* and *y* in this way, their inner product *ζ*_*ω*_(*x*) *ζ*_*ω*_(*y*) is an unbiased estimator of *k*(*x*,*y*). It is proved that $\zeta_{\omega }(x)=\sqrt{2}cos(\omega^{\prime }x+b)$ satisfy the Eq (13) where *ω* is the random projection direction drawn from *p*(*ω*) and *b* is bias term drawn uniformly from [0, 2π]. Thus, *Z*(*x*) can be defined as Eq (14) where D is the number of random projection directions. Combing the Eq (13) and Eq (14), we have the Eq (15), description of approximating calculation for kernel evaluation *k*(*x*,*y*).

$$Z(x)=\sqrt{\frac{1}{D}}\sum_{j=1}^{D}\zeta_{\omega_{j}}(x)$$ (14)

k(x,y)=Z(x)Z(y) (15)

In this paper, the random Fourier features were used to construct an explicit feature map *Z*(*x*) in order to transform the extracted features into a linearly separable space. After the kernel mapping layer, a new mapped feature vector is obtained that could be separated by a linear model, thus the output layer is just a fully-connected layer with softmax as activation function whose output is the probability distribution over classifications.

## Experiment

### Data distribution

14 stocks were randomly selected in the Chinese A-share market covering the period from 2016-11-01 to 2020-03-31. The training data includes the period from 2016-11-01 to 2019-10-31 and the test data includes the period from 2019-11-01 to 2020-03-31. The label distribution of training data and test data was shown in Table 2. The label distribution is balanced in training data and test data, which indicates that the model after training will not be biased to any label.

**Table 2:** Label distribution of datasets.

	Positive	Negative
Training data	0.5220	0.4780
Test data	0.5297	0.4703

### Comparative models

Firstly, in order to assess the usefulness integrating the technical analysis and sentiment analysis, the results of the proposed RCNK model were compared with the models adopting only time series data or sentiment data as input. Secondly, the comparative experiment with convolutional neural kernel (CNK) model [36] was carried out to evaluate the impact of treating the sentiment data as time series data. The CNK model, which contained no LSTM layer, did not consider the temporal features of sentiment data. But it had the same explicit kernel mapping layer with the RCNK model. Finally, the performance of RCNK model and recurrent convolutional neural network (RCNN) model [28, 29] were compared to test the effectiveness of explicit kernel mapping layer. Compared with RCNK model, the RCNN model had no explicit kernel mapping layer. It used several full-connected layers in the classification section of the model like traditional neural networks. For brevity, abbreviations in Table 3 identified those models mentioned. The same training data was used to train different models.

**Table 3:** Abbreviations for different models.

Abbreviation	Description
RCNK	Recurrent convolutional neural kernel model
RCNK-T	RCNK model only with time series data as input
RCNK-S	RCNK model only with sentiment data as input
CNK	Convolutional neural kernel model in [36]
RCNN	Recurrent convolutional neural network model in [28,29]

### Model performance metrics

To evaluate the performance of different models, three metrics were used: accuracy, Matthews Correlation Coefficient (MCC) and accumulated return. The accumulated return is the difference between the final account value and the initial account value after a period of trading simulation. The other two metrics measured the predictive power of the model. Accuracy describes the ability of the model to correctly predict all classes of labeled data. MCC ∈ [–1, 1] is usually used for measuring the quality of binary classification, even if the label distribution in datasets is unbalanced [38]. It is essentially a coefficient describing the correlation between real labels and predicted labels. It means the perfect prediction when the value is 1. These two metrics are computed as:

$$accuracy=\frac{TP+TN}{TP+FN+FP+TN}$$ (16)

$$MCC=\frac{TP\times TN-FN\times FP}{\sqrt{\left(TP+FP)\times (TP+FN)\times (TN+FP)\times (TN+FN\right)}}$$ (17)

where TP is the number of correctly predicted positive samples; TN is the number of correctly predicted negative samples; FN is the number of positive samples that were predicted to be negative; FP is the number of negative samples that were predicted to be positive.

### Hyper parameter selection

Before the experiment, two main issues must be settled. The first one is the selection of the time frame parameter which is also called input window length for text data. As it was assumed before, the influence of sentiment on the stock price would last for a period and gradually recede over time. This hyper parameter explains how long the emotional impact lasts.

**Fig 5:**
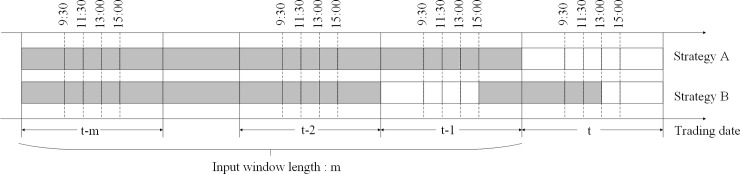
Two strategies for collecting posts.

The second one is the time slot selection for text data. The stock market is open from 9:30 to 11:30 in the morning and from 13:00 to 15:00 in the afternoon. In previous studies, most researchers chose the posts of *m* whole days before transaction date *t* to predict the stock price movement of transaction date *t*. This was called strategy A. But Kim and Kim [39] found that investor sentiment was positively affected by prior stock price performance and doubted its predictive power. This finding reminds us to pay attention to the immediacy feature of posts in the message board. It was observed that many topics about predicting the current direction of the stock price trend were posted in current trading time whereas more topics about predicting the trend of the next trading day were posted after the stock market closed. Therefore, another strategy B to select the time slot is to consider the posts in current trading date *t* and the posts after trading time at date *t*−1. To have an insight on this process, two strategies for selecting the time slot are showed in Fig 5. The shaded area in Fig 5 is the time slot during which the posts were collected.

The real stock trading was simulated by simplifying the trading strategy proposed by [40]. The initial account value was set to be RMB 10,000 and the transaction cost was ignored. For strategy A, if the model predicts the stock close price of the next trading date will go up, the trader will hold or buy the stock at the open price of current date. Otherwise the trader will sell the stock at the open price of the current date. For strategy B, if the model predicts the stock close price of the current date will go up, the trader will hold or buy the stock at the open price of the current date. Otherwise the trader will sell the stock at the open price of the current date.

**Fig 6:**
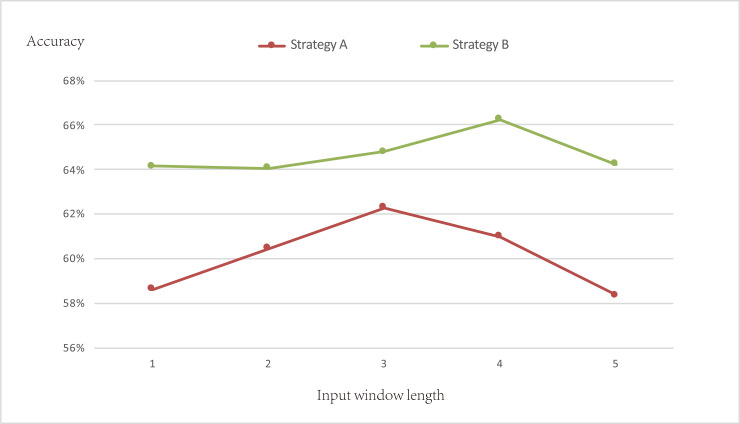
Accuracy of two strategies with different input window length.

It is of same importance to predict the positive samples and the negative samples correctly because the results directly affect whether we will buy or sell stocks. Thus, the accuracy was chosen to evaluate the model with different input window lengths. The average prediction accuracies of 14 stocks with different input window length are showed in Fig 6. It is found that the prediction accuracy of strategy B is generally higher than that of strategy A, which proves that the posts in message boards really have immediacy feature. Adding part of the posts in current trading date to inputs and adopting appropriate trading strategy can help improve prediction accuracy and accumulated returns. Though the most optimal value of input window length for two strategies are different, the prediction accuracy reaches highest within three days before the transaction date, which further illustrates the sentiment extracted from message board should be treated as sequential data and it is appropriate for predicting the short-term stock price movement.

After determining the most optimal value of input window length, trade simulation was conducted with two strategies. The results are shown in Table 4. Average accumulated returns of strategy B are higher than that of strategy A. Both metrics indicate that the model with strategy B performs better. Hence, it is chosen to conduct experiment in next section.

**Table 4:** Average accumulated returns for two strategies with different input window length.

	Input window length				
	1	2	3	4	5
Strategy A	732	1660	2910	1724	648
Strategy B	3947	3833	3701	4244	3886

## Results and discussion

We test our proposed model and other comparative models. The performance of MCC was consistent with that of accuracy. The detailed experimental results are shown in Table 5. When evaluating trading simulation, we compared these models with simple Buy & Hold strategy which reflected the individual stocks price movement and can be treated as the baseline. The results are shown in Table 6. From the results, we can learn that:

**Table 5:** Predictive performance of different models.

Metrics	Models				
	RCNK	RCNK-T	RCNK-S	CNK	RCNN
Accuracy	66.26%	59.44%	63.25%	65.61%	63.66%
MCC	0.3918	0.2747	0.3572	0.3613	0.3111

**Table 6:** Accumulated returns of individual stocks with different models and buy & hold strategy.

Stocks	Models					Buy & Hold strategy
	RCNK	RCNK-T	RCNK-S	CNK	RCNN	
000005	997	723	-248	554	529	-894
000060	154	751	-355	683	-780	-1172
000068	3098	-1077	1759	2408	2101	-2518
000157	2907	1382	-52	1466	3765	188
000338	5376	1884	4647	5459	4297	433
000425	6776	4554	6382	6661	7919	1422
000547	8380	203	8902	7647	10697	3878
000630	-710	-632	233	166	-442	-787
000751	-732	-187	-702	-704	-287	-1016
000876	7525	572	5750	6222	4716	3202
000921	3060	-612	2662	2708	3839	-1555
000998	5138	1274	6278	11099	7277	3784
002299	5963	-65	2778	2624	3031	-1689
002714	11479	-53	4218	9642	3871	1848
Average	4244	623	3018	4045	3609	366

1. RCNK model performed better than RCNK-T model and RCNK-S model. This indicated that these two data sources (the financial time series data and posts in the message board) complemented each other, and the model with two data sources as inputs can achieve better results in stock price movement prediction. It is noteworthy that RCNK-S model performed much better than RCNK-T model. This is likely caused by the inputs of two models containing different amount of information. The public posts contain much explicit and predictive sentiment data which directly indicates whether users think the stock price will rise or fall. It is much easier to model the correlation between the explicit sentiment data and the trend of stock price movement. Whereas the historical price data and technical indicators do not contain explicit information for prediction, it needs more sophisticated model to extract implicit features for prediction. Our proposed RCNK model involves only one CNN layer and one LSTM layer, which means it is a shallow network and may not work well when the input contains too many implicit and abstract features.
2. RCNK model was superior to CNK model. The difference between RCNK model and CNK model is that the former regards sentiment data of several trading days as sequential data but the latter only accumulates them together. It indicated that the temporal feature of sentiment information did exist and can effectively improve the performance of the model.
3. Results of RCNK model outperformed that of RCNN model which had no kernel mapping layer, which proved the effectiveness of the explicit kernel mapping technique.
4. Though not every stock made profits in trading simulation, our proposed RCNK model achieved the most average accumulated return. It performed much better than baseline (Buy & Hold strategy). This is mainly because even in a long-term falling stock market, it can still make short-term profits by good prediction.

**Fig 7:**
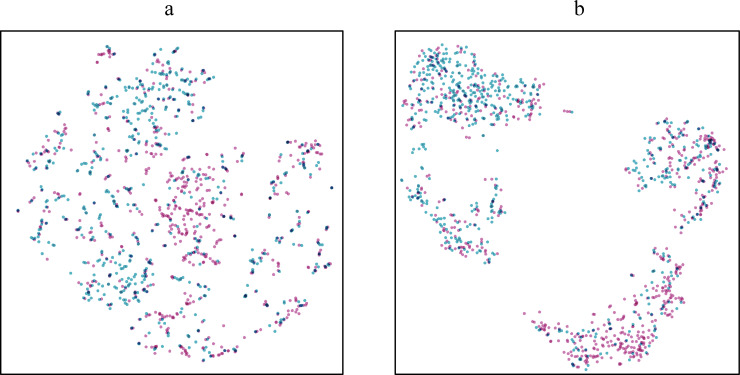
The t-SNE projections of test data. (a) The t-SNE projections of the test data after computation of LSTM layer; (b) The t-SNE projections of the test data after computation of explicit kernel mapping layer.

In order to demonstrate the effectiveness of explicit kernel mapping layer more intuitively, the outputs of LSTM layer and explicit kernel mapping layer were fetched separately and were projected into two-dimensional space. Explicit kernel mapping layer utilized the random Fourier features to construct an explicit mapping function *Z*(*x*) that can map the outputs of LSTM layer into a higher dimensional feature space. The t-SNE visualization results of the fetched outputs were shown in Fig 7. The points with different colours refer to the samples with different labels after computation. It can be seen that the classification plane of the outputs from the explicit kernel mapping layer is clearer and more linear than that from LSTM layer. The experiment results also showed that the explicit kernel mapping technique can help to improve the prediction accuracy.

## Conclusions

In this paper, a model named Recurrent Convolutional Neural Kernel (RCNK) model was proposed for stock price movement prediction. In order to improve the prediction accuracy of the model and the accumulated returns of trading simulation, the RCNK model was optimized from three aspects: data collection, text data processing and classifier of the model. The main contributions of this study can be summarized as following:

1. The financial time series data and posts in stock message board were used as two data sources for extracting complementary features. The proposed model combined the advantages of technical analysis and sentiment analysis. It learned sentiment embeddings with temporal features from posts in stock message board as well as financial embeddings from financial time series data. The word embedding technique, which considered semantic features as well as structural features of text data, was utilized as data representation method. It performed better than that with a single analysis.
2. It was assumed that the impact of public mood on the stock price would last for a period and gradually recede over time. Thus, the posts were treated in stock message board as sequential text data and long-term and short-term memory neural network (LSTM) was used to extract temporal features. This technique does help improve the prediction accuracy in stock price movement.
3. The explicit kernel mapping layer was used to replace serval full-connected layers in traditional deep learning models. Random Fourier features were used for constructing explicit mapping function which could project the input into a high-dimensional space. This approach is an approximate computation of the kernel method. It reduced the parameter of the model and the risk of overfitting. In this way, the proposed model bridged the deep learning model and kernel method. It showed better performance in prediction accuracy and accumulated returns compared with other deep learning models in the same dataset.

When selecting hyper parameters for model, it was found that the accumulated return and accuracy of the model were inconsistent. That indicated high accuracy of the model did not mean high profit. Thus, how to evaluate the performance of the model comprehensively is an interesting research direction for future work. From the result of trading simulation, we can see that some individual stocks fall in a long term. If we want to get good returns in the real stock market, we need not only good prediction for stock price movement but also portfolio optimization and better trading strategies. Therefore, portfolio optimization and trading strategies are worthy of further study. Besides, except for the financial series data and sentiment data, there are still many factors influencing the movement of the stock price. Integrating more sources of data and extracting various features for stock price movement prediction is worth further study.
